# MOF-based stimuli-responsive controlled release nanopesticide: mini review

**DOI:** 10.3389/fchem.2023.1272725

**Published:** 2023-09-12

**Authors:** Shuhui Hu, Chang Yan, Qiang Fei, Bo Zhang, Wenneng Wu

**Affiliations:** ^1^ Food and Pharmaceutical Engineering Institute, Guiyang University, Guiyang, China; ^2^ Shanghai Engineering Research Center of Green Energy Chemical Engineering, College of Chemistry and Materials Science, Shanghai Normal University, Shanghai, China

**Keywords:** nanopesticide, controlled release, stimuli-responsive, metal-organic frameworks, bioactivity

## Abstract

By releasing an adequate amount of active ingredients when triggered by environmental and biological factors, the nanopesticides that respond to stimuli can enhance the efficacy of pesticides and contribute to the betterment of both the environment and food safety. The versatile nature and highly porous structure of metal-organic frameworks (MOFs) have recently garnered significant interest as drug carriers for various applications. In recent years, there has been significant progress in the development of metal-organic frameworks as nanocarriers for pesticide applications. This review focuses on the advancements, challenges, and potential future enhancements in the design of metal-organic frameworks as nanocarriers in the field of pesticides. We explore the various stimuli-responsive metal-organic frameworks carriers, particularly focusing on zeolitic imidazolate framework-8 (ZIF-8), which have been successfully activated by external stimuli such as pH-responsive or multiple stimuli-responsive mechanisms. In conclusion, this paper presents the existing issues and future prospects of metal-organic frameworks-based nanopesticides with stimuli-responsive controlled release.

## 1 Introduction

Pesticides have an indispensable function in enhancing the quality and productivity of crops, as well as fulfilling the increasing food demands of the world’s population. According to Reichenberger and others (Reichenberger et al., 2007), the global production of pesticides has reached 4.6 million tons per year. However, over 90% of these pesticides end up in the environment and agricultural products, with less than 0.1% effectively targeting pests. According to Hussain and Kah (Kah and Hofmann, 2014; Hussain et al., 2019), nanopesticides have advantages in enhancing the scattering, durability, and effectiveness of pesticides on the intended subjects. Additionally, they aid in the attachment, longevity, and penetration of pesticides while minimizing their residual contamination in non-target regions and the surroundings.

By responding to particular microenvironmental stimuli, the release of pesticides can be controlled intelligently and accurately, leading to improved drug effectiveness and decreased toxic and adverse effects, as demonstrated by Ahmadi and Sun (Sun et al., 2014; Ahmadi et al., 2020).The innovation of pesticide formulations has gained more attention due to the emergence of smart pesticide delivery systems (PDS) utilizing nanomaterials (Sun et al., 2014; Ahmadi et al., 2020; Zhang et al., 2020; Song et al., 2021; Wu H et al., 2021; Wu W et al., 2021; Zhou et al., 2021). Selecting intelligent nanocarriers, such as permeable nanomaterials, plays a crucial role in enhancing the effectiveness of nano-pesticide compositions (Lawson H. D et al., 2021; Wang et al., 2022a; Ma et al., 2023). Metal-organic frameworks (MOFs) are a novel type of porous nanomaterials characterized by their well-organized crystalline framework structures. These frameworks possess distinctive attributes, such as a well-arranged composition, remarkable surface area, and substantial pore volume, which facilitate the adsorption of functional molecules on their outer surface or within their interconnected channels. This capability allows for the entrapment of these molecules within the framework (Furukawa et al., 2013; Wang S et al., 2020; Mallakpour et al., 2022). Furthermore, rational design and control of organic ligands, metal nodes, and synthesis conditions enable the flexible regulation of MOFs structure and associated properties, catering to specific application scenarios (Qu et al., 2022; Wang et al., 2022b). In particular, for the altered MOFs, it has the potential to facilitate the attainment of controlled release of pesticides in response to stimuli (Dong et al., 2021). Furthermore, metal-organic frameworks (MOFs) have shown significant promise in the fields of biomedicine and pesticide usage due to their limited ability to accumulate within the body (Teplensky et al., 2019; Qiangqiang et al., 2020; Mahmoudi et al., 2022). Even though much attention has been paid to the development of MOF preparation methods and their application in pesticide adsorption, loading, and detection (Huiping et al., 2021; Rojas et al., 2022; Chenyu et al., 2023), in this review, we mainly focus on the latest advancements in the creation of controlled release nanopesticides utilizing MOFs, particularly remarkable progress has been made in the research of stimulus-responsive controlled release nanopesticides using ZIF-8 as the carrier.

## 2 Characteristics and preparation of MOFS-based environmental-responsive nanopesticides

Different types of stimuli-responsive nanopesticides are developed using MOFs: 1) Active ingredients (AIs) are encapsulated by MOFs through the one-pot method, resulting in stimuli-responsive nanopesticides like ZIF-8-based pH-responsive delivery nanoparticles. This prevents premature losses in the environment and ensures specific release of β-Cypermethrin in acidic environments associated with termite infestation pesticide (Ma et al., 2022a). 2) Initially, MOFs are fabricated to encapsulate pesticides, which are then coated with special materials like zain to facilitate stimuli-triggered pesticide release (Ma et al., 2022b). 3) An integrated pesticide-fertilizer system is constructed by combining MOF with hollow mesoporous silica and polydopamine, enabling simultaneous encapsulation of multiple pesticides and provision of micronutrient Zn^2+^ (Ji et al., 2021). 4) MOF-based nanopesticides are formed by directly using pesticide molecules as organic ligands and combining them with metal ions. For example, 2D-MOF can be prepared by chelating the herbicide glufosinate and Cu^2+^ (Sierra-Serrano et al., 2022). The above MOFs can be tailored to respond to microenvironmental stimuli, such as pH, redox, enzymes to achieve precision agriculture. Moreover, most MOFs materials are pH-responsive.

## 3 Progress of MOFS-based environmentally responsive nanopesticides

### 3.1 Application of ZIF-8 in stimulus-responsive nanopesticides

Two primary techniques utilized in the synthesis of MOFs are the solvothermal approach (Hu et al., 2015) and the non-solvothermal technique (Lawson S et al., 2021). The one-pot method, as a typical of non-solventothermal method, has series of advantages of containing simple in operation, mild reaction conditions, which facilitate ZIF-8 preparation.

ZIF-8, a well-known MOF that is responsive to stimuli, is composed of a zeolitic imidazole acid skeleton consisting of Zn^2+^ and 2-methylimidazolate. According to Zhang, Sun and Saliba (Saliba et al., 2018; Sun et al., 2019; Zhang W et al., 2022), ZIF-8 decomposes in acidic conditions due to the dissociation of coordination bonds at pH 5.0–6.0. Consequently, it can serve as a carrier material that responds to acidity, as mentioned by Chen, Zhang and Mejías. (Chen et al., 2021; Mejías et al., 2021; Zhang X et al., 2022). Significantly, ZIF-8 releases zinc ions upon breakdown, which enhance plant development, establishing it as an eco-friendly carrier material in contrast to MOFs that contain toxic metals like copper, zirconium, and chromium (Yan et al., 2017; Yang J et al., 2017). Consequently, ZIF-8 exhibits great potential as an intelligent pesticide delivery system.

**FIGURE 1:**
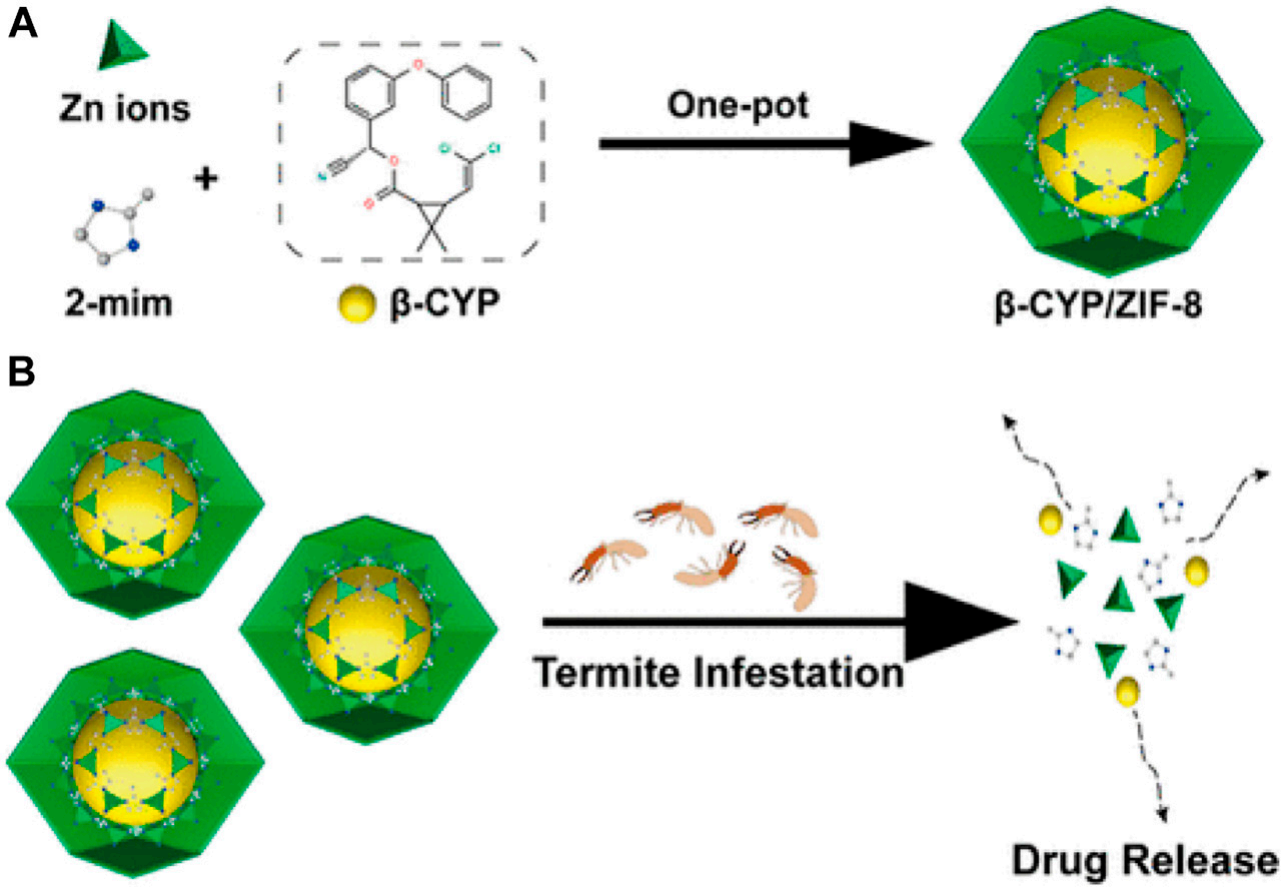
**(A)** The one pot method was employed for the synthesis of β-CYP/ZIF-8, **(B)** which subsequently facilitated the targeted drug release in response to termite infestation (Ma et al., 2022b).

Harmful organisms such as termites and *Staphylococcus griseus* can produce a slightly acidic environment, which enables controlled release of pesticides by degrading ZIF-8. The study conducted by Ma and others, β-CYP/ZIF-8 was synthesized in a single step according to Ma’s method (Ma et al., 2022a), as shown in Figure 1. The micro-acidic environment, in which termites flourish, can enhance the release of β-CYP through the decomposition of ZIF-8, leading to improved targeting of pesticides and reduced toxic side effects (Sun et al., 2019). This favorable acidic environment supports termite life and enhances the specific release. Furthermore, β-CYP/ZIF-8 diminished the harmful impacts on organisms that are not the intended target. Therefore, the nanoformulations prepared using the one-pot technique hold immense promise for utilization in both the sustainable control of pests and the preservation of the environment.

**FIGURE 2:**
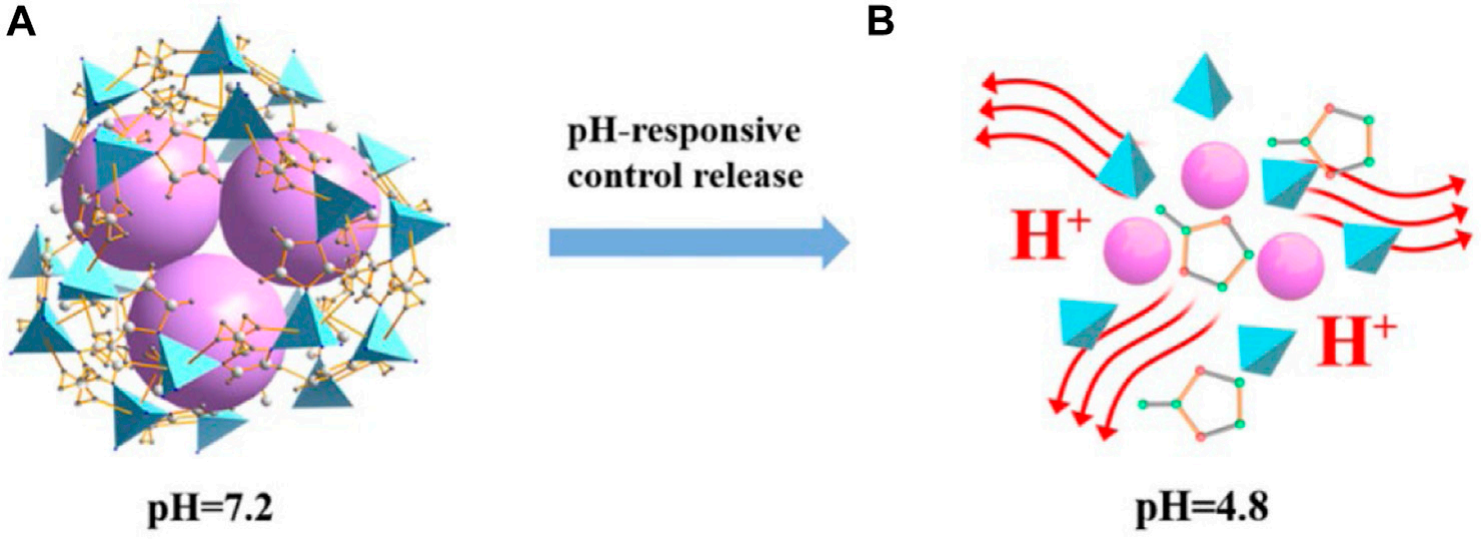
The release of DZ@ZIF-8 is controlled by the pH response (Ren et al., 2022).

Likewise, a site-specific nano-release system containing dazomet (DZ@ZIF-8) was synthesized using ZIF-8 through a one-pot approach (Ren et al., 2022). Due to the production of acid compounds come from *Botrytis cinerea*, caused the breakdown of the DZ@ZIF-8 framework and the subsequent localized release of DZ (Figure 2). Additionally, the utilization of DZ@ZIF-8 can prevent the harmful effects of DZ on plants, enabling its use throughout the growth of plants. Consequently, employing ZIF-8 as a nanocarrier enhanced the efficiency of pesticide utilization while mitigating its harmful side effects.

**FIGURE 3:**
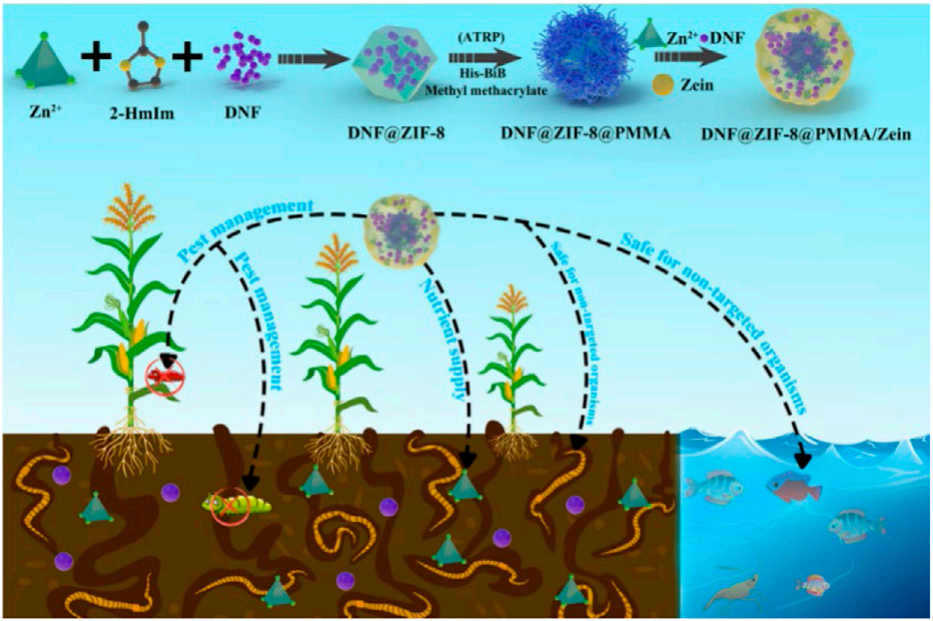
Utilization and implementation plan of the MOF composite (DNF@ZIF-8@PMMA/Zein) for delivering pesticides and fertilizers (Ma et al., 2022b).

**FIGURE 4:**
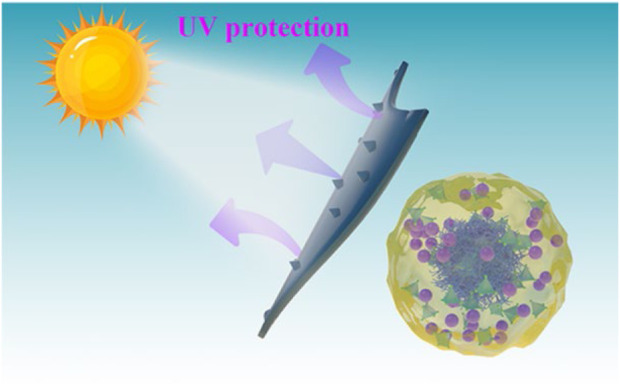
The possible mechanism for the MOF hybrid’s ability to protect against ultraviolet radiation (Ma et al., 2022a).

The study conducted by Ma and others. In their study, Ma (Ma et al., 2022a) developed a hybrid material (DNF@ZIF-8@PMMA/zein) that responds to changes in pH and is triggered by protease. This material was designed for delivering dinotefuran (DNF), as shown in Figure 3. ZIF-8 was initially fabricated for encapsulating DNF, and then hydrophobic poly (methyl methacrylate) (PMMA) was polymerized *in situ*. In the end, an additional coating of zein is applied to the outer layer in order to aid in the release of cargo triggered by protease. The UV resistance of DNF was enhanced by nearly 10 times (Figure 4) and pest control efficiency was significantly improved when using the nano formulation in comparison to free DNF. This hybrid MOF can be targeted against pests and diseases and has promising applications in sustainable agriculture.

**FIGURE 5:**
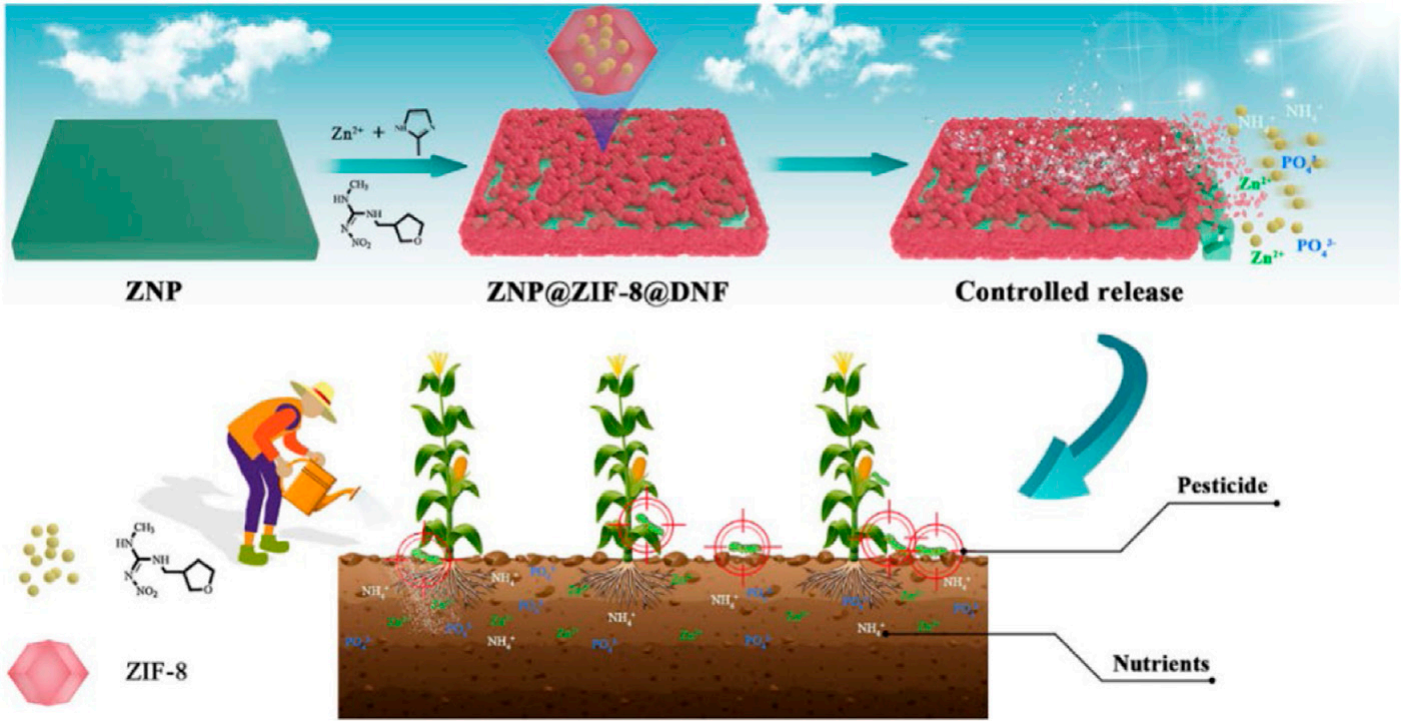
This stimuli-response contains a depiction of the creation of a slow-release pesticide-fertilizer combination (ZNP@ZIF-8@DNF) that responds to pH, along with its possible implementation in the field of sustainable farming (Ma et al., 2021).

An intriguing development in the field involved the creation of a pesticide-fertilizer blend that is eco-friendly. This blend utilized ammonium zinc phosphate (ZNP) and *in situ* synthesized ZIF-8 as sources of nutrients, along with dinotefuran (DNF) as the pesticide (Ma et al., 2021). Instead of being loaded through additional adsorption, DNF was enclosed *in situ* while the ZIF-8 synthesis process took place, resulting in enhanced stability and prevention of premature or rapid release. Figure 5 illustrates the pH-sensitive sustained release characteristic of the water-repellent ZIF-8. The system combining pesticide and fertilizer had notable impacts on pre-cultivation of corn seeds, cultivation of soil, and control of pests.

**FIGURE 6:**
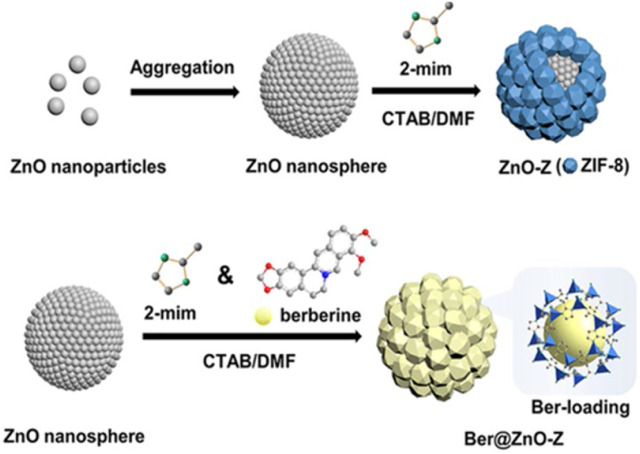
Preparation of ZnO-Z and Ber@ZnO-Z nanosphere depicted in the illustration (Liang et al., 2022a).

Using ZnO nanomaterials as Zn source, ZnO-ZIF-8 (ZnO-Z) nanocomposite carriers with core-shell structure can be prepared using *in situ* crystal growth strategy. ZnO-Z, a pH-responsive core-shell nanocarrier, was prepared by Liang and others (Liang et al., 2022), using ZnO nanosphere as the core and ZIF-8 as the shell. Figure 6 illustrates the formation of nano-formulation Ber@ZnO-Z, where the berberine (Ber) was incorporated. In an acidic environment, Ber@ZnO-Z has the ability to quickly release Ber, which is consistent with the pH of the soil where the occurrence of tomato bacterial wilt disease is frequent. By binding to DNA, RNA and proteins, Ber inhibits DNA replication, RNA transcription, and protein synthesis, rendering it a secure medication that does not induce drug resistance (Li et al., 2018). Zinc oxide and titanium dioxide have been widely used in various applications due to their unique properties. ROS production can be triggered by ZnO, MgO, and CuO (Applerot et al., 2009; Applerot et al., 2012; Cai et al., 2018), consequently, ZnO-Z also exhibits a specific antimicrobial impact. Hence, this task holds significance in managing bacterial diseases that affect the soil.

### 3.2 Application of Fe-based MOFs in stimuli-responsive nanopesticide

Fe-based MOFs, particularly MIL-101(Fe) and MIL-101(Fe)-NH_2_, are frequently employed in the field of agriculture. Crop growth relies on iron, a crucial micronutrient that plays a vital part in photosynthetic reactions (Armin et al., 2014). Insufficient iron levels can significantly impact plant development and metabolic processes, including chlorophyll production, nitrogen fixation, and respiration (Bindra et al., 2019). Moreover, Fe-based MOFs are utilized as an iron fertilizer to augment plant growth. (Anstoetz et al., 2016; Abdelhameed et al., 2019).

MIL-101(Fe) is composed of Fe_3_O_4_ and 1,4-benzenedicarboxylate (BDC) (Horcajada et al., 2010; Dong et al., 2017). It possesses a high surface area, extensive porosity, and favorable biocompatibility (Lebedev et al., 2005; Wu and yang, 2017), making it suitable as a carrier for pesticide loading. Nevertheless, the premature release of pesticide occurs in Fe-based MOFs nanoparticles (Shan et al., 2020). The Fe-based MOFs surface underwent modification to attain its responsive characteristics to stimuli, including particular triggers within cells (e.g., the redox). The disintegration of the Fe-based MOFs framework structure was induced by, leading to the stimuli-responsive behavior of cargoes (Wang et al., 2022a).

**FIGURE 7:**
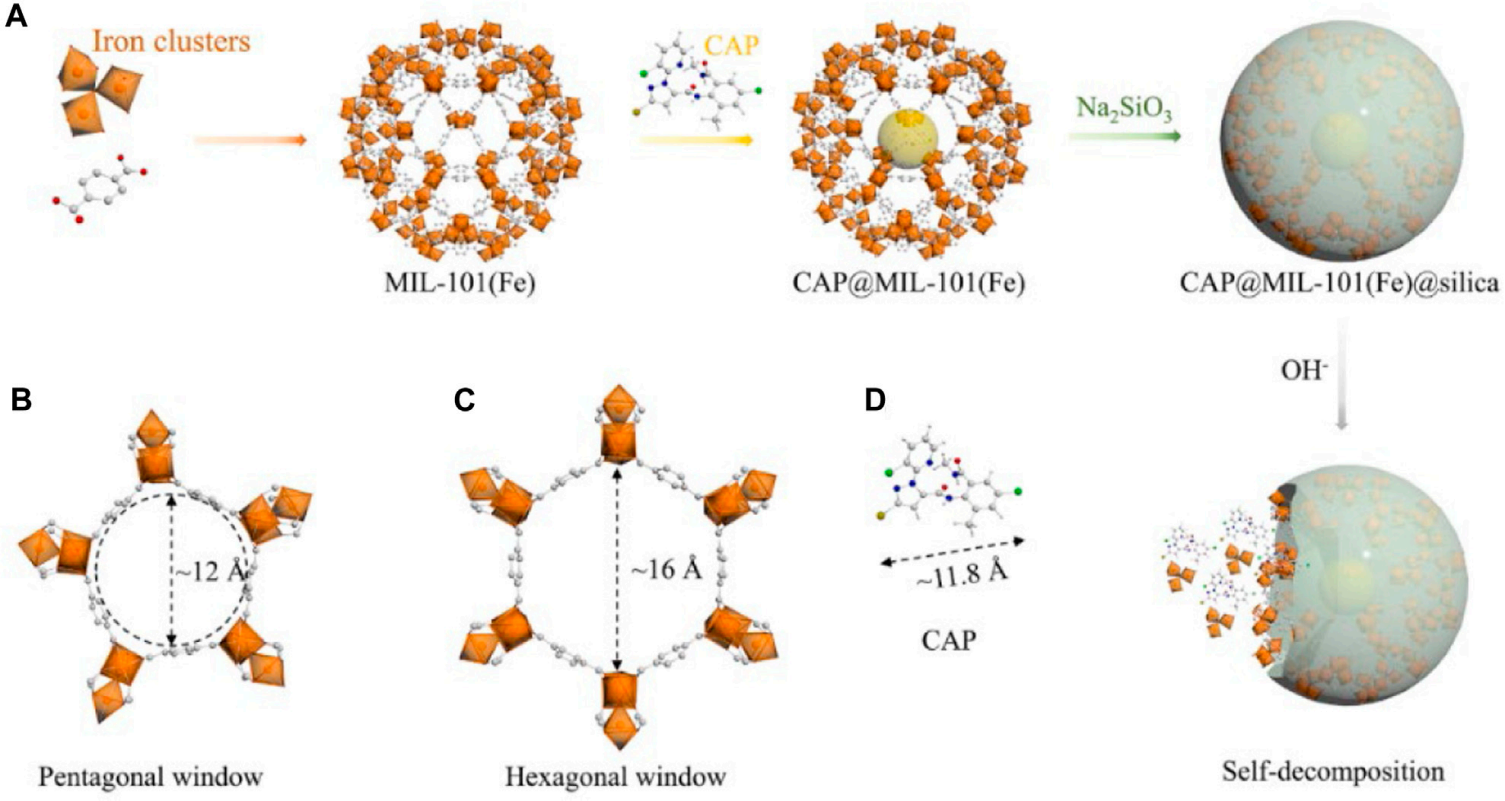
The scheme outlines the process of preparing and potentially releasing CAP@MIL-101(Fe)@silica **(A)**. The pentagonal window **(B)**, hexagonal window **(C)**, and CAP molecule **(D)** are shown in a ball-and-stick view with free dimensions (Å) (Gao et al., 2021).

Alkaline pH-responsive properties are exhibited by MOFs formed by combining high-valent metal ions with ligands based on carboxylic acid, such as PCN-222 and MIL-101. This is due to the self-decomposition of the carboxylic acid-based backbone in alkaline conditions, as mentioned in the studies by Taylor-Pashow and Yuan (Taylor-Pashow et al., 2009; Yuan et al., 2018). Additionally, Fe-based MOFs have the potential to serve as a fertilizer, enhancing the growth of plants (Anstoetz et al., 2016; Abdelhameed et al., 2019). The authors Gao and others (Gao et al., 2021) developed a clever nanocarrier called MIL-101(Fe)@silica that served two purposes: targeted delivery and plant nourishment. This nanocarrier effectively transported chlorantraniliprole (CAP) for eco-friendly pest control, leading to enhanced UV resistance and insecticidal efficacy against small cabbage moth larvae. Figure 7 illustrates the potential process of formation and the mechanism of release. SiO_2_ can undergo hydrolysis to form soluble silanols (≡Si-OH) in the presence of alkali catalysis (Yang G.Z et al., 2017). In addition, lepidopteran pests that feed on plants have a highly basic digestive system (with a pH of up to 12), which serves as a mechanism for the controlled release of CAP (Gao et al., 2019; Wang X.G et al., 2020). Furthermore, MIL-101(Fe) and silica have the potential to serve as sources of iron (Fe) and silicon (Si) for enhancing plant development (El-Shetehy et al., 2021). This work offers promising strategies for efficient pesticide application and precise control of pests.

**FIGURE 8:**
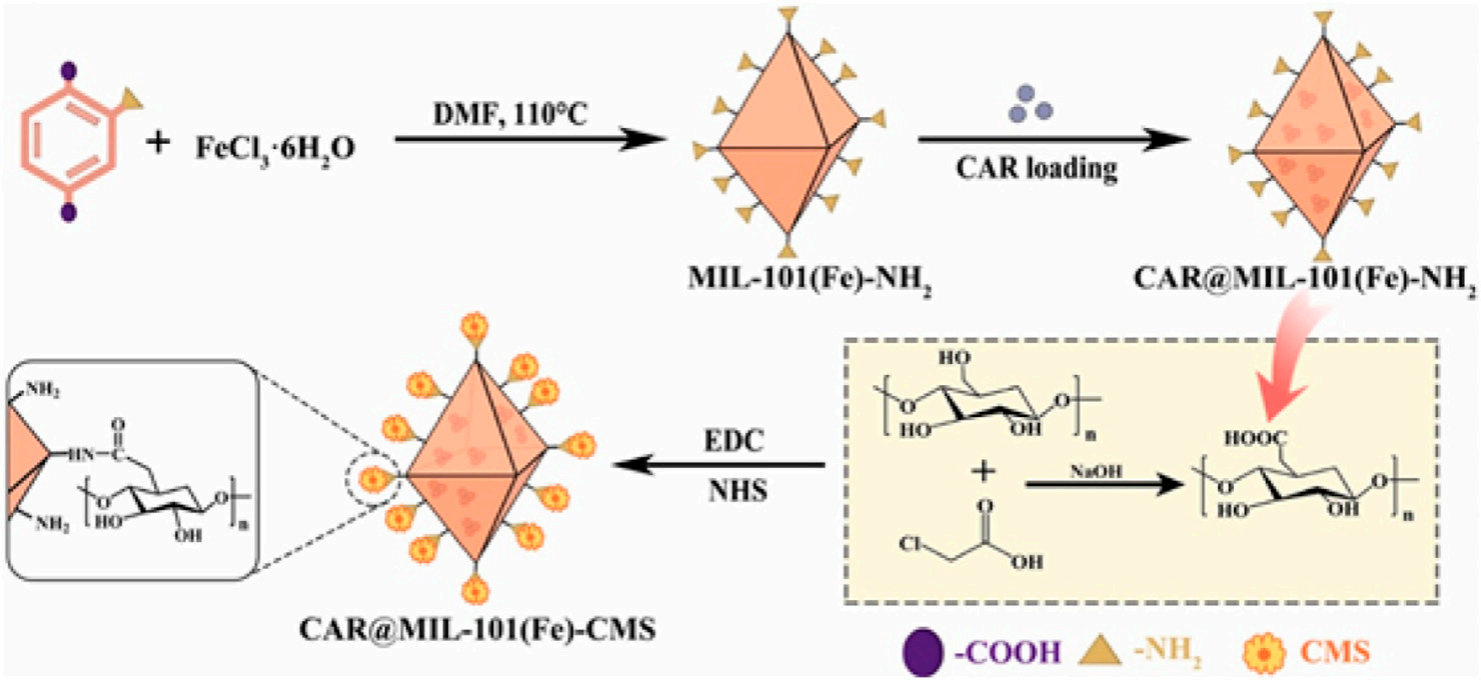
The method for creating CAR@MIL-101(Fe)-CMS nanoparticles (Liang et al., 2022).

Liang and others (Liang et al., 2022b) utilized carboxymethyl starch (CMS) in conjunction with MIL-101(Fe)-NH_2_ containing chlorantraniliprole (CAR) to create a nano-release system CAR@MIL-101(Fe)-NH_2_-CMS, which responds to pH, redox, and α-amylase stimuli (Figure 8). Carboxymethyl starch, a cheap polymeric carbohydrate composed of glucose units connected by glucose bonds, can be broken down by alpha-amylase in the insects' midgut (Holtof et al., 2019; Yu et al., 2021; Siemińska-Kuczer et al., 2022). The alkaline gut of insects contains both α-amylase and glutathione, which play a role in breaking down ingested substances. This enables CAR@MIL-101(Fe)-CMS nanoparticles to break apart in the insect intestine and promptly release CAR for precise targeting, as demonstrated by Khandelwal, Sharifloo and Banerjee et al.(Khandelwal et al., 2016; Sharifloo et al., 2016; Banerjee et al., 2022). By combining the digestive mechanism of bugs with the response-regulated discharge technology of insecticides, an efficient approach to pest control is achieved.

Liang and others (Liang et al., 2022a) synthesized PYR@FeMOF-pectin by incorporating pectin into MIL-101(Fe)-NH_2_ along with pyraclostrobin (PYR), resulting in a composite material exhibiting dual responsiveness to redox and pectinase stimuli. Pectin, being a natural polymer, constitutes a significant portion of plant cell walls and possesses favorable water solubility and biocompatibility (Broxterman and Schols, 2018; Mohammadinejad et al., 2019). Moreover, it offers distinct benefits compared to alternative substances in agricultural practices (Bellemjid et al., 2021). Moreover, the pectin’s carboxyl group can undergo cross-linking with the amino amide of MIL-101(Fe)-NH_2_, as stated by Shitrit and co-workers (Shitrit et al., 2019). Consequently, when plants are infected, pectinases secreted by plant pathogenic microorganisms swiftly degrade it. On the other hand, *P. aeruginosa* possesses a robust antioxidant defense mechanism that generates glutathione during host plant infection. This glutathione helps in neutralizing reactive oxygen species produced by the host defense response. The design of PYR@FeMOF-pectin nanopesticide is accurately tailored to the specific surroundings in which it operates, resulting in a precise delivery of PYR that targets (Kubicek et al., 2014; Samalova et al., 2014; Chiniquy et al., 2019; Sabbadin et al., 2021), as demonstrated earlier.

### 3.3 Other types of MOFs in stimuli-responsive nanopesticides

Agriculture can benefit from the promising potential of various other MOFs. Cobalt-imidazole metal-organic framework (MOF) ZIF-67 is produced by combining cobalt nitrate hexahydrate and 2-methylimidazole, possessing a larger pore size compared to ZIF-8. This larger pore size makes it a more suitable option as a carrier and delivery platform for pesticides, as stated by Tsalaporta and others (Tsalaporta and MacElroy, 2020). The authors Zhang and others (Zhang W et al., 2022) synthesized a carrier-based acetamiprid with pH sensitivity by utilizing ZIF-67. The pH-responsive release of acetamiprid occurs because of the ZIF-67’s weak acidic group. Early in the infestation of host plants, griseus produces acidic substances such as oxalic acid, which facilitates the rapid disintegration and release of acetamiprid (Zhang X et al., 2022). The study not only showcases the potential of ZIF-67 as a nanocarrier for effectively managing gray mold, but also enhances the conventional approach to addressing agricultural diseases.

**FIGURE 9:**
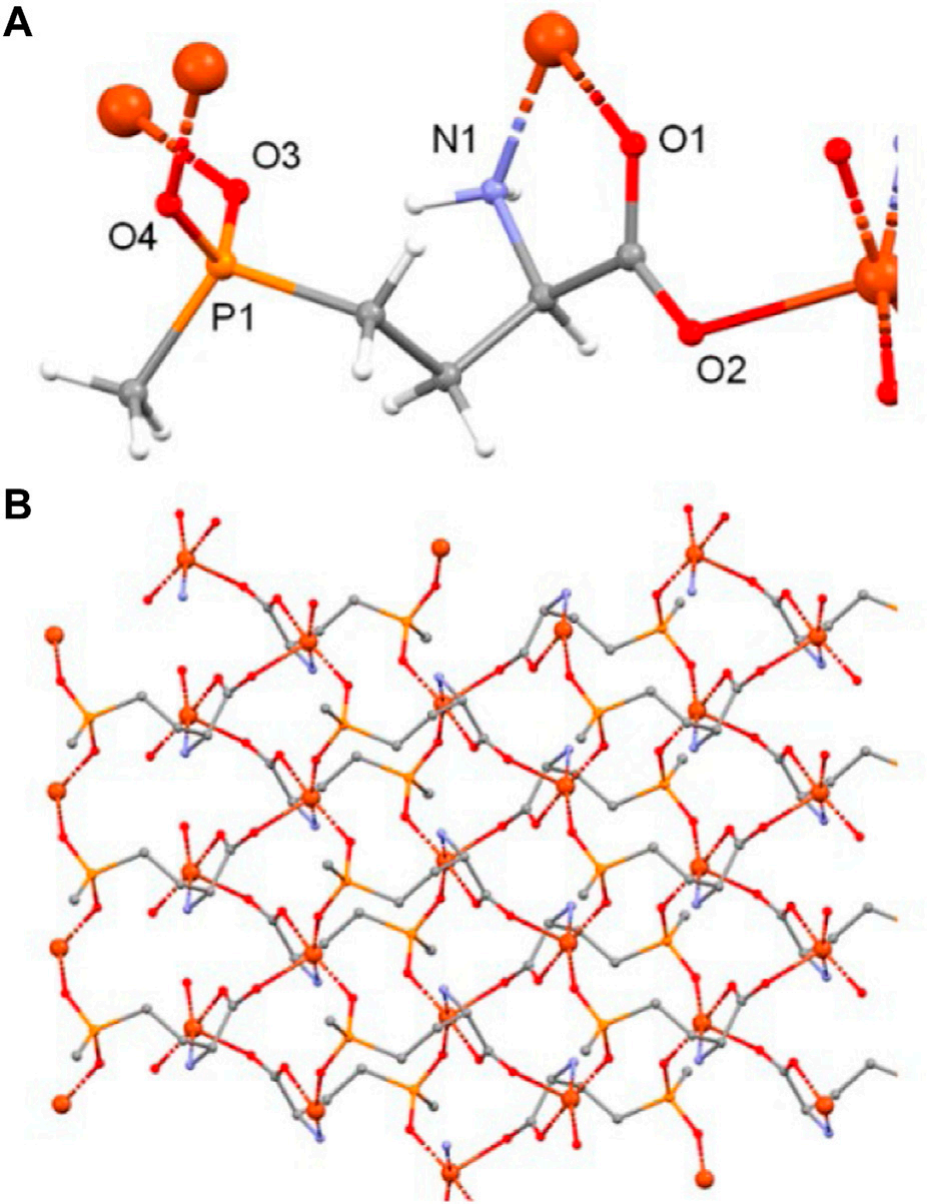
**(A)** The GR-MOF-7 crystal structure reveals the coordination mode of the glufosinate ligand, which binds to four Cu^2+^ ions through all of its donor atoms, **(B)** Additionally, a view of the sheet is shown along the a crystallographic axis. To ensure clarity, the hydrogen atoms have been excluded (Sierra-Serrano et al., 2022).

Creating multifunctional agrochemicals shows promise through the synthesis of MOFs using herbicide and antimicrobial components. Sierra-Serrano and others prepared a kind of Cu-based MOFs (2D-MOF), called GR-MOF-7, using glufosinate and Cu^2+^ (Sierra-Serrano et al., 2022) (Figure 9). The findings indicated that GR-MOF-7 exhibits outstanding antimicrobial efficacy against *Staphylococcus aureus* and *Escherichia coli*. Furthermore, GR-MOF-7 employs pesticides as ligands during MOF synthesis, thereby creating opportunities for the secure and effective utilization of MOF in the field of agriculture.

**FIGURE 10:**
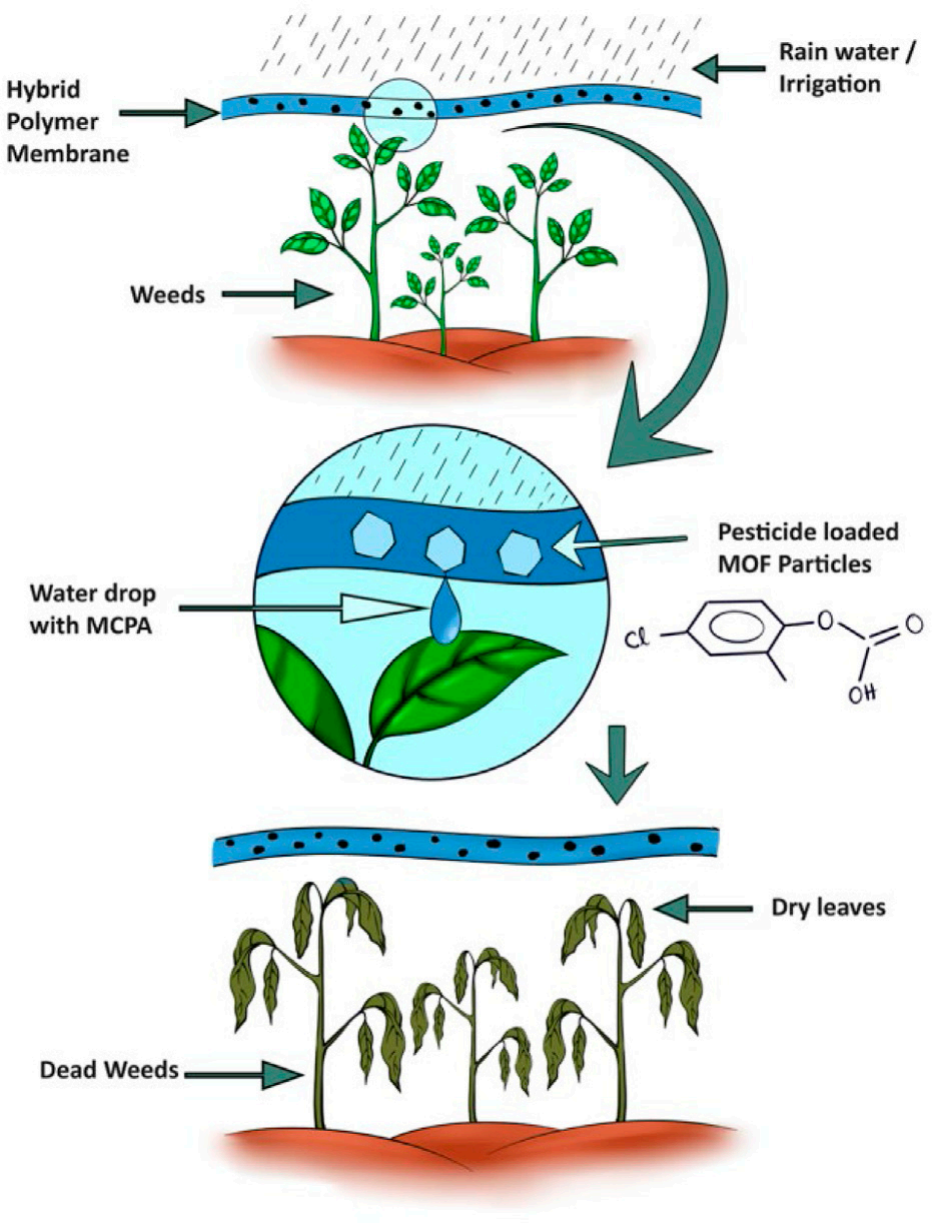
The illustration showcases a composite membrane made of polymer-MOF, which enables the delivery of pesticides in direct contact with weeds. This delivery is made possible through the rainwater or irrigation system (Mahmoud et al., 2022).

According to Song and others (Song et al., 2022), UiO-66 is a zirconium-based MOF that exhibits excellent durability and a notable rate of loading. Intelligent nanocarriers with better performance, such as UiO-66 derivatives include UiO-66-NH_2_, UiO-66-(COOH)_2_. Mahmoud and others (Mahmoud et al., 2022) obtained the amino or carboxyl groups by introduction. In order to enhance biodegradability, Mahmoud and others incorporated 2-methyl-4-chlorophenoxyacetic acid (MCPA) into various zirconium-based MOFs (UiO-66, UiO-66-NH_2_, and UiO-67). During the synthesis of MOFs, MCPA is either loaded *in situ* or loaded afterwards. Subsequently, the MOFs loaded with MCPA are integrated into composite membranes made of biodegradable polycaprolactone (PCL) (Figure 10). The completion of research experiments has identified various concentrations that can effectively impede the growth of different plant species at various stages. This discovery suggests that these composites hold great potential for future agricultural applications.

The authors of the song and others, in their study, Song and others (Song et al., 2022) developed pH-sensitive PDSs by utilizing carboxymethyl starch (CMC)-modified UiO-66-NH_2_, a zirconium-based organic framework, as a nanocarrier for acetamiprid (ATP).The surface area of UiO-66-NH_2_-CMC is extensive and it contains numerous pores, enabling efficient loading of ATP at a rate of 90.79%.The release behavior of ATP@UiO-66-NH_2_-CMC was responsive to changes in pH. In a separate investigation, researchers utilized the temperature-responsive polymer N-isopropyl acrylamide (PNIPAm) that had been altered with UiO-66-(COOH)_2_ to serve as a transporter for the highly effective insecticide indoxacarb (IDC). The PNIPAm polymer chains underwent contraction as the temperature surpassed the lower critical solution temperature (LCST), resulting in the nanoparticles collapsing. This collapse, as reported by Karakasyan and Guo (Guo and Gao, 2007; Karakasyan et al., 2008), led to a notable enhancement in the release rate of IDC. Due to its affordability, uncomplicated preparation process, effectiveness, and safety, this approach shows great potential for advancing pesticide formulation innovation and plant protection (Mahmoud et al., 2022; Song et al., 2022; Wan et al., 2022).

## 4 Summary and outlook

Due to their abundant functional properties, MOFs nanomaterials have found extensive applications in diverse fields. By utilizing them as carriers for pesticides and incorporating them with changes in microenvironmental stimulation (such as pH, redox, light, and other conditions) caused by the interaction among pests (such as pests, weeds, molds, etc.). This review provides a summary of the benefits, release mechanism triggered by stimuli, and bioactive characteristics of ZIF-8 and FeMOFs as carriers in the development of nanopesticides with stimulus-responsive properties. These carriers enable the intelligent and targeted release of pesticides, leading to enhanced utilization and efficiency in pest control while significantly mitigating environmental pollution. Furthermore, MOFs-based nanopesticides possess not only the characteristics of pesticides, but also can also function as nutrients for crop growth. This integrated pesticide-fertilizer approach offers substantial benefits in agriculture. Nevertheless, the present study encounters two issues. Firstly, the investigation on the absorption and transportation of MOF nanomaterials in plants remains limited, and the assessment of MOF materials' safety is still incomplete. Therefore, it is crucial to maintain focus on future toxicological and safety studies. Secondly, the current encapsulation rate of the majority of pesticides loaded with MOFs is inadequate. Enhancing the encapsulation rate of specific pesticides holds immense importance for the effective utilization of MOF pesticides. Moving forward, our focus will be on advancing MOFs nanopesticides and enhancing the encapsulation rate. Additionally, we will dedicate further resources to enhancing the safety evaluation system. To sum up, the utilization of MOF nanocarriers offers significant benefits in achieving a prompt release of pesticides when stimulated, thereby promoting the advancement of 'intelligent pesticides.
